# Use of Psychoactive Substances Before Incarceration Among Prison Inmates With Drug Abuse or Dependence: Data From the OPPIDUM Program

**DOI:** 10.1111/fcp.70058

**Published:** 2025-12-12

**Authors:** Zeinab Abbas, Clémence Lacroix, Elisabeth Jouve, Céline Eiden, Joelle Micallef, Hélène Peyrière, and the French Addictovigilance network, Alexandre Peyre, Reynald Leboisselier, Celian Bertin, Nathalie Fouilhe, Anne‐Sylvie Caous, Johan Thiery, Marylène Guerlais, Lauriane Charuel, Stéphanie Pain, Emilie Jouanjus

**Affiliations:** ^1^ Centre d'Addictovigilance, Centre Hospitalier Universitaire Montpellier France; ^2^ Centre d'Addictovigilance Paca‐Corse Service de Pharmacologie Clinique et Pharmacosurveillance, APHM‐Sainte Marguerite, Amu, Ins, Inserm UMR1106 Marseille France; ^3^ PCCEI, University of Montpellier, INSERM, University of Antilles, CHU Montpellier Montpellier France; ^4^ Centre d'Addictovigilance Centre Hospitalier Universitaire Bordeaux France; ^5^ Centre d'Addictovigilance Centre Hospitalier Universitaire Caen France; ^6^ Centre d'Addictovigilance Centre Hospitalier Universitaire Clermont‐Ferrand France; ^7^ Centre d'Addictovigilance Centre Hospitalier Universitaire Grenoble France; ^8^ Centre d'Addictovigilance Centre Hospitalier Universitaire Lille France; ^9^ Centre d'Addictovigilance Centre Hospitalier Universitaire Nancy France; ^10^ Centre d'Addictovigilance Centre Hospitalier Universitaire Nantes France; ^11^ Centre d'Addictovigilance Centre Hospitalier Universitaire Paris France; ^12^ Centre d'Addictovigilance Centre Hospitalier Universitaire Poitiers France; ^13^ Centre d'Addictovigilance Centre Hospitalier Universitaire Toulouse France

**Keywords:** addictovigilance, OPPIDUM program, prison inmates, psychoactive substances

## Abstract

**Background:**

The objective of this study was to assess the prevalence of dependence and abuse of psychoactive substances (PAS) among prison inmates, using data from the OPPIDUM program between 2013 and 2022.

**Methods:**

OPPIDUM is an annual, cross‐sectional national program, conducted among users consulting in specialised addiction centres. Prison inmates were questioned about their PAS use during the week preceding their incarceration. Two groups of participants were compared: prison inmates who reported simple use of PAS and those with abuse/dependence problems.

**Results:**

A total of 2626 individuals responded to the program (men, 91.6%; mean age, 34.4 ± 9.30 years), reporting 5352 PAS. The main PAS consumed were cannabis (52.8%), cocaine/crack (28.6%), benzodiazepines (23.1%) and heroin (14.8%). Opioid substitution treatment (OST) was reported by 54.9% of participants. Several variables were associated with a significantly increased odds of abuse/dependence: intravenous use (OR, 4.608; 95% CI, 1.44–14.69; *p* = 0.01), PAS illegal acquisition (OR, 3.79; 95% CI, 2.19–6.58; *p* < 0.0001), heroin/speedball use (OR, 4.24; 95% CI, 1.16–15.48; *p* = 0.029) and cocaine/crack use (OR, 3.3; 95% CI, 1.47–7.39; *p* = 0.004). Conversely, being on OST protocol was associated with a lower odds of abuse/dependence (OR, 0.511; 95% CI, 0.28–0.93; *p* = 0.028).

**Limitations and Conclusion:**

The main limitations of the study include self‐reported PAS use without objective diagnoses, sometimes incomplete data on PAS use and incarceration and a sample biased toward inmates linked to substance abuse services, which likely overestimates the prevalence of PAS use. However, these results highlight the importance of assessing factors associated with substance abuse and dependence for appropriate prevention and management among prison inmates.

**Abbreviations**
FAN: French Addictovigilance Network
OST: opioid substitution treatment
PAS: psychoactive substances
SUD: substance use disorders
WHO: World Health Organization

## 1 Introduction

In 2019, there were over 856 000 people in prisons located in the 27 EU Member States including 70 818 in France [1]. Substance use disorders (SUD) among offenders as well as the association between crime and substance use have been a persistent issue for assessment in correctional settings [2]. Prison inmates around the world detain large numbers of individuals with SUD, which increases the risk of mortality after prison release as well as repeat offending [3]. In this latter study, the pooled prevalence estimate of SUD in male prison inmates was 30% [3].

It was reported that prison inmates who have been imprisoned are more likely to use or have used psychoactive substances (PAS) and experience drug‐related problems [1], with nearly half of all people jailed in the US experiencing problematic substance use prior to incarceration [4]. In a recent Belgian study, 54% of participants used PAS in the 12 months prior to their incarceration, mainly cannabis (40.9%), cocaine/crack (27.5%), amphetamines (23.9%) and heroin (13.8%) [5]. Moreover, in a recent French study, most of the prison inmates interviewed described consuming PAS before their detention, sometimes at higher levels compared to those observed in the general population [6]. The use of PAS before incarceration can continue in prison; in fact, 1 in 10 prison inmates in France recounts the use of illicit PAS (cocaine, crack, MDMA, heroin), and 1 in 4 sought to inject a substance at least once since incarceration [6].

Only a few countries in Europe have a comprehensive national system that captures and understands the nature of drug use, drug‐related problems, interventions and treatment provided within custodial environments [1]. In France, the OPPIDUM (Observatoire des produits psychotropes Illicites ou Détournés de leur usage médicamenteux/Observation of Illegal Drugs and Misuse of Psychotropic Medications), an annual, cross‐sectional national program (repeated each year in October since 1990 and based on the French Addictovigilance Network [FAN]), collects anonymous data on PAS consumed by patients recruited in drug dependence care centres including prison medical centres [7, 8]. By characterizing the modalities of PAS use, this study makes it possible to identify risky uses. It also makes it possible to identify simple uses and abuse/dependence.

This study aims to estimate the prevalence of dependence and/or abuse of PAS use in prison inmates 1 week before incarceration and to assess the factors impacting the prevalence of simple use versus abuse or dependence.

## 2 Methods

According to the World Health Organization (WHO), a PAS is defined as any substance which, due to its chemical nature, disrupts the functioning of the central nervous system (sensations, perceptions, moods, feelings, motor skills) or which modifies states of consciousness [9]. These substances include psychotropic medications (benzodiazepines, opioids), legal (alcohol, nicotine) or illegal substances (heroin, cocaine, etc.)

This manuscript was prepared in accordance with the STROBE reporting guideline for observational cross‐sectional studies.

### 2.1 Data Source

The OPPIDUM, one of the epidemiological tools of the FAN, is an annual, cross‐sectional program that, every year in October, collects anonymous data on PAS consumed by patients recruited in drug dependence care centres. The FAN brings together 13 centres located in the French administrative regions and aims to monitor the risks and consequences of PAS with abuse potential including prescription drugs and other legal and illegal substances (except alcohol and tobacco) [10]. Patients are seen in substance abuse treatment facilities, e.g., addiction departments (hospitalisation, consultation), addiction treatment centres, harm reduction centres and prison medical centres. These voluntary centres are selected by the regional addictovigilance centre, according to their ability to regularly participate in the program. Eligible patients are those with drug abuse or dependence (as defined by the Diagnostic and Statistical Manual of Mental Disorders, Fourth Edition [American Psychiatric Association, 1994] criteria) or receiving opiate maintenance treatment (OST), a clinically supervised treatment that replaces illicit or misused opioids with prescribed, long‐acting opioid agonists (methadone or buprenorphine) to manage opioid dependence and reduce associated harms [7, 11]. About 5000 subjects are included in the survey each year.

### 2.2 Study Population

In France, each prison has a medical centre comprising doctors, pharmacists and nurses who receive prison inmates needing care and/or medications. Participation in the OPPIDUM program is offered to each eligible patient seen in these units during the month of data collection. Patients with only alcohol or tobacco consumption were excluded.

### 2.3 Data Collection

Data were collected using the anonymised OPPIDUM questionnaire, through face‐to‐face interviews with participants [7]. The OPPIDUM questionnaire is in French. In this study, data were extracted from the OPPIDUM database between 2013 and 2022.

The questionnaire consisted of two parts:

1. The patient form included data on sociodemographic characteristics such as age, gender, pregnancy, living as a couple, raising children, education, employment, living in stable accommodations and financial resources. The first section also included data related to four reported addictive behaviours including alcohol dependence, tobacco consumption, first substance used and first substance leading to an addiction (except tobacco and alcohol), current opioid substitution treatment and medication used for opioid substitution.
2. The PAS form included information related to reported PAS consumed during the week preceding the incarceration for this context. For each substance, the following variables were collected: name of the substance, pharmaceutical form, route(s) of administration, frequency of intake, average quantity of daily doses, dose increment within last 6 months, withdrawal symptoms, concomitant alcohol use, duration of consumption, consumption as part of an abuse or dependence and means of acquisition. Other data were computed from the questionnaire but not directly disclosed by participants and included using doses higher than the daily dose recommended by the Summary of Product Characteristics (SPC) and doses over twice the maximum daily dose recommended by the SPC. The occurrence of withdrawal symptoms was also described by participants and answers were grouped as yes or no.

### 2.4 Primary and Secondary Outcomes

The primary outcome of this study was to identify the factors associated with higher odds of abuse and dependence among prison inmates during the study period. The secondary outcome was to determine the progress of trends and types of PAS used over the years, via descriptive analysis.

### 2.5 Statistical Analysis

Substance abuse and substance dependence were defined by DSM‐IV criteria [12]. Two groups of participants were identified: “simple use” (simple use is occasional or regular consumption that does not cause damage at the somatic, psychoaffective and/or social levels) and the second group that included prison inmates reporting “abuse” (abuse is characterised by consumption leading to damage detected at the somatic, psychoaffective and/or social levels) or “dependence” (denoted by an irrepressible need to consume, called craving). Prison inmates for whom this information (simple use or abuse/dependence) was not available were excluded from the analysis. Tobacco and alcohol use were included as confounding variables to control for their effects but were not part of the main independent group classifications, which separated participants into “simple use” and “dependence” based on other criteria. This allowed the analysis to focus on the primary variables while adjusting for tobacco and alcohol influences.

Descriptive statistics were used to examine the participant demographic characteristics and the OPPIDUM answers. Frequencies and proportions or percentages were used to describe all characteristics. A bivariate analysis was performed to identify the variables to be included in the multivariate analysis model and only variables with a *p* < 0.15 were included in the model. The Student's *t*‐test and one‐way ANOVA were used for comparison of categorical independent variables and the Kruskal–Wallis test was applied when Levene's test was significant. For continuous variables, the Pearson or Spearman test was deployed in the absence of normal distribution.

Based on the results of the bivariate analysis, a multivariate logistic regression was used to evaluate the relationship between reported abuse or dependence and independent variables (age groups, minor participants, living as a couple, stable accommodation, level of education, alcoholic dependence, tobacco use, age at first consumption, age of use of first substance inducing dependence, substitution protocol, frequency of consumption, oral or sublingual use, inhaled route of administration, injected drug use, smoking, start of consumption, mode of acquisition by donation, mode of acquisition from dealer, mode of acquisition by multiple prescriptions, mode of acquisition by theft, illegal acquisition, dose increment, effect, concomitant use of alcohol, withdrawal symptoms).

All statistical analyses were carried out using the Statistical Package for Social Sciences (SPSS Inc., Chicago, IL) Version 23, and *p* < 0.05 was considered significant.

All variables were tested using bivariate analysis to identify significant correlation and reliable variables to be incorporated into the final multivariate analysis. Variables with *p* < 0.15 were then included in the final model. Yet, four variables were also excluded due to the high percentage of missing values (>20%): mode of acquiring substances, obtaining substances using a falsified prescription, doses higher than the authorised dose and doses two times higher than the authorised dose. The choice of 20% as a cutoff was motivated by balancing the need to minimise bias due to incomplete data against the desire to retain as much information as possible from our dataset.

Finally, the following variables were included in the final multivariate logistic regression: age groups, living as a couple, education level, alcoholic dependence, tobacco use, age of first use, age of first use causing dependence, substitution protocol, reported substance, frequency of use, oral route of administration, inhaled route of administration, nasal route of administration, intravenous route of administration, start of consumption, obtaining substance by deal, obtaining substance by donation, obtaining substances by theft, obtaining substances by multiple prescriptions, illegal obtaining of substances, dose increment, desired effect, concomitant use of alcohol and craving after discontinuation.

### 2.6 Ethical Approval

The OPPIDUM programme only collects anonymous data. No ethics approval is requested to process anonymous data according to French law. All information was treated as strictly confidential.

## 3 Results

A total of 2626 participants were interviewed between 2013 and 2022 reporting consumption of 5352 PAS. After removal of product records where abuse/dependence or simple use was not specified, only 3674 product records were included in the analysis corresponding to a total of 2116 participants. The population was mainly represented by men (92.05%). The average age was 33.6 ± 9.1 years. Demographic characteristics of all included participants in the study are reported in Table 1.

**TABLE 1:** Characteristics of included participants.

		Total sample, *N* (%)	Subjects with criteria of abuse/dependence/simple use not documented	Total: simple use + abuse/dependence, *N* (%)	Subjects with only simple use, *N* (%)	Subjects with at least one PAS with abuse/dependence, *N* (%)	*p*
Total number of participants		2626	510	2116	375 (14.2%)	1741 (85.8%)	
Age		34.43 + 9.30	37.9 ± 9.2	33.6 ± 9.1	35.1 ± 8.5	33.25 ± 9.2	<0.0001
Sex	Female	218/2602 (8.4%)	51/504 (10.1%)	167/2098 (7.95%)	29/371 (7.8%)	138/1727 (8%)	0.911
	Male	2384/2602 (91.6%)	453/504 (89.9%)	1931/2098 (92.05%)	342/371 (92.2%)	1589/1727 (92%)	
Living as a couple		920/2581 (35.6%)	178/495 (35.9%)	742/2086 (35.6%)	110/370 (29.7%)	632/1716 (36.8%)	0.01
Professional activity		804/2459 (32.7%)	170/475 (35.8%)	634/1987 (31.9%)	112/356 (31.5%)	522/1631 (32%)	0.842
Child in charge		717/2558 (28.0%)	176/488 (36.1%)	541/2070 (26.1%)	99/368 (26.9%)	442/1702 (26%)	0.712
Stable accommodation		1544/2454 (62.9%)	299/466 (64.2%)	1245/1988 (62.6%)	206/350 (58.9%)	1039/1638 (63.4%)	0.108
Level of education	Primary	321/2527 (12.7%)	55/483 (11.4%)	266/2044 (13%)	35/363 (9.65%)	231/1681 (13.7%)	0.063
	Secondary	1701/2527 (67.3%)	310/483 (64.2%)	1391/2044 (68%)	247/363 (68.05%)	1144/1681 (68.1%)	
	Bachelor	407/2527 (16.1%)	98/483 (20.3%)	309/2044 (15.2%)	62/363 (17.1%)	247/1681 (14.7%)	
	High school	98/2527 (3.9%)	20/483 (4.1%)	78/2044 (3.8%)	19/363 (5.2%)	59/1681 (3.5%)	
Resources	Financial insecurity with social income	1100/2477 (44.4%)	238/476 (50%)	862/2001 (43.1%)	157/351 (44.7%)	705/1650 (42.7%)	0.609
	Severe financial insecurity	609/2477 (24.6%)	90/476 (18.9%)	519/2001 (25.9%)	93/351 (26.5%)	426/1650 (25.8%)	
	Regular income	768/2477 (31.0%)	148/476 (31.1%)	620/2001 (31%)	101/351 (28.8%)	519/1650 (31.5%)	
Alcohol dependence		803/2572 (31.2%)	102/499 (20.4%)	701/2073 (33.8%)	97/369 (26.3%)	604/1704 (35.5%)	0.001
Tobacco use		2479/2588 (95.8%)	475/497 (95.6%)	2004/2091 (95.8%)	339/368 (92.1%)	1665/1723 (96.6%)	<0.0001
Substances route of administration	Intravenous	187/2626 (7.1%)	8/510 (1.6%)	179/2116 (8.5%)	8/375 (2.1%)	171/1741 (9.8%)	<0.0001
	Nasal	782/2626 (29.8%)	33/510 (6.5%)	749/2116 (35.4%)	56/375 (14.9%)	693/1741 (39.8%)	<0.0001
	Inhaled	394/2626 (15%)	14/510 (2.7%)	380/2116 (17.9%)	20/375 (5.3%)	360/1741 (20.7%)	<0.0001
Illegal obtaining of at least 1 PAS		1944/2583 (75.3%)	90/489 (18.4%)	1854/2093 (88.6%)	220/369 (59.6%)	1634/1725 (94.7%)	<0.0001
Illegal obtaining of medications		583/1771 (32.9%)	50/469 (10.6%)	488/1302 (37.5%)	52/288 (18.05%)	436/1014 (43.0%)	<0.0001
Age of first substance use (mean ± SD)		16.08 + 5.0	17.55 ± 6.2	15.7 ± 4.65	16.4 ± 5.1	15.6 ± 4.5	0.037
Age of first substance inducing dependence (mean ± SD)		19.2 + 5.9	21.3 ± 6.6	17.95 ± 5.66	19.6 ± 5.4	18.4 ± 5.6	0.698
Opioid maintenance protocol		1439/2621 (54.9%)	474/508 (93.3%)	965/2113 (45.6%)	243/373 (65.1%)	722/1740 (41.5%)	<0.0001
Opioid maintenance medications	Methadone	696/1439 (48.4%)	214/474 (45.1%)	482/965 (49.9%)	113/243 (46.5%)	369/722 (51.1%)	
	Buprenorphine	740/1439 (51.4%)	259/474 (54.6%)	481/965 (49.8%)	130/243 (53.5%)	351/722 (48.6%)	0.314
	Morphine	3/1439 (0.2%)	1/474 (0.2%)	2/965 (0.2%)	0 (%)	2/722 (0.3%)	
Doses higher that the daily dose recommended by the SPC		182/1037 (17.6%)	10/251 (3.9%)	172/786 (21.90%)	30/215 (14%)	142/571 (24.9%)	0.001
Dose twice higher that the daily dose recommended by the SPC		42/1037 (4%)	2/251 (0.79%)	40/786 (5.1%)	4/215 (1.9%)	36/571 (6.3%)	0.011
Concomitant alcohol use		970/2626 (36.9%)	80/510 (15.7%)	890/2116 (42%)	80/375 (21.3%)	810/1741 (46.5%)	<0.0001
Polysubstance user		1544/2626 (58.8%)	52/510 (10.2%)	1492/2116 (70.5%)	291/375 (77.6%)	1201/1741 (69.0%)	0.001
Withdrawal symptoms		1591/2626 (60.6%)	252/510 (49.4%)	1339/2116 (63.3%)	154/375 (41%)	1185/1741 (68%)	<0.0001
Substance used	Morphine	42/2626 (1.59%)	3/510 (0.59)	39/2116 (1.8%)	2/375 (0.5%)	37/1741 (2.1%)	0.038
	Heroin or speedball	389/2626 (14.8%)	13/510 (2.5%)	376/2116 (17.8%)	15/375 (4.0%)	361/1741 (20.7%)	<0.0001
	Benzodiazepines	607/2626 (23.1%)	17/510 (3.33%)	590/2116 (27.9%)	163/375 (43.5%)	427/1714 (24.5%)	<0.0001
	Cannabis	1388/2626 (52.8%)	39/510 (7.45%)	1349/2116 (63.8%)	165/375 (44%)	1184/1741 (68.0%)	<0.0001
	Cocaine or crack	750/2626 (28.6%)	23/510 (4.5%)	727/2116 (34.4%)	48/375 (12.8%)	679/1741 (39%)	<0.0001
	Crack only	80/2626 (3%)	2/510 (0.39%)	78/2116 (3.7%)	6/375 (1.6%)	72/1741 (4.1%)	0.018
	Cocaine only	677/2626 (25.8%)	21/510 (4.1%)	656/2116 (31.0%)	43/375 (11.5%)	613/1741 (35.2%)	<0.0001

The *p* value was calculated between both groups: simple use versus abuse/dependence.

Abbreviation: SPC, summary of product characteristics.

When asked for the first PAS used, cannabis was the first reported substance in comparison to all other substances that showed a much lower percentage over the period. In addition, cannabis was also the most reported substance first inducing dependence followed by heroin and then cocaine.

### 3.1 Reported Substances Used During the Week Before Incarceration

**FIGURE 1:**
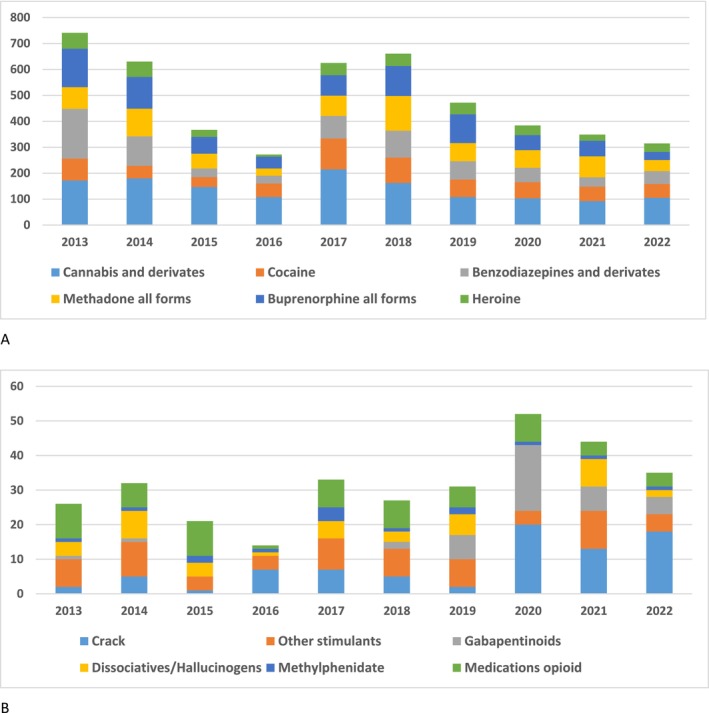
Reported substances 1 week before incarceration (*n* = 5352). A, Main reported substances; B, less reported substances.

As shown in Table 1, the main PAS reported in the total population were cannabis (52.8%), cocaine (25.8%), benzodiazepines (23.1%) and heroin (14.8%). Figure 1 describes the evolution of the number of PAS reported by participants over the study period. Cannabis was the most reported substance over all years, followed by cocaine, benzodiazepines and then heroin. The prevalence of the last three substances overlapped during the first 3 years. We identified consumption trends: increased crack use from 2020, peak consumption of gabapentinoids (mainly pregabalin) in 2020, decreased use of buprenorphine as an OST offset with methadone from 2020. New psychoactive substances (NPS) represented only one case and concerned a hallucinogenic substance, 5‐methoxy‐*N*‐methyl‐*N*‐isopropyltryptamine (5‐MEO‐MIPT).

Concomitant alcohol use was reported for 36.9% of the included subjects. The percentage of concomitant alcohol use was lower among participants on OST, compared to those not on OST: 28% versus 47%, *p* < 0.0001.

**FIGURE 2:**
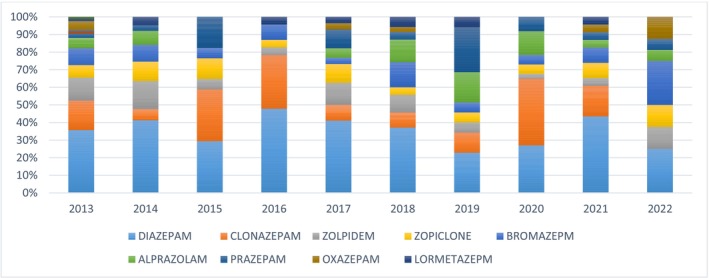
Evolution of the benzodiazepines reporting during the study period (*N* = 445). All benzodiazepines with less than five reported cases during the study period were not included in the comparison table.

In our study, we assessed the change in reported benzodiazepine use over the study period (Figure 2). Diazepam was the most used benzodiazepine in most years. A substantial increase in reported prazepam use was observed in 2019 compared to other years; in that year, it was the most used benzodiazepine.

### 3.2 Assessment of Abuse and Dependence Versus Simple Use

Table 1 represents the main characteristics of the two groups of subjects in the study. Participants reporting simple use were significantly older (35.1 versus 33.25 years old). Prison inmates with abuse/dependence issues consumed significantly more PAS, whether medications or illicit substances but also tobacco and alcohol, and considerably more often presented criteria for diversion and abuse: diverted route of administration—intravenous (9.8% versus 2.1%), nasal (39.8% versus 14.9%), inhaled (20.7% versus 5.3%); illegal acquisition (94.7% versus 59.6%). Surprisingly, frequency of polysubstance use was higher in the simple use group (69.5% versus 77.6%).

### 3.3 Results of Multivariate Analysis

Several variables were identified in the final model of the multivariate analysis showing significant association with dependence and abuse versus simple use. Living as a couple, using doses higher than the recommended dose, injection drug use, intranasal use, illegal acquisition, benzodiazepines, heroin or speedball, cannabis and cocaine (or crack) use as well as experiencing withdrawal symptoms from at least one of the substances were all associated with a higher odds of dependence or abuse compared to simple use. Injection drug use was associated with four times higher odds of abuse and dependence with an OR of 4.608 (95% CI [1.445; 14.69], *p* < 0.0001). Similarly, intranasal route use was associated with a twofold increase in the odds of abuse and dependence with an OR of 2.1.09 (95% CI [1.113; 3.994], *p* = 0.022). Among the substances reported by participants, heroin or speedball demonstrated the highest odds of substance dependence and abuse, followed by cocaine or crack, when compared to substances such as cannabis and benzodiazepines. Specifically, heroin/speedball use was associated with significantly elevated odds (OR, 4.241; 95% CI [1.161–15.486], *p* = 0.029), while cocaine/crack use also showed a strong association (OR, 3.301; 95% CI [1.474–7.392], *p* = 0.004).

On the other hand, being on an OST was associated with lower odds of abuse and dependence on substance use in general among participants (OR, 0.511; 95% CI [0.280; 0.931], *p* = 0.028).

All results of the multivariate analysis are reported in Table 2.

**TABLE 2:** Multivariate analysis.

	*p*	OR Bivariate	95% CI for EXP(B)		*p*	OR Multivariate	95% CI for EXP(B)	
			Lower	Upper			Lower	Upper
Living as a couple	0.010	1.378	1.080	1.758	0.013	1.764	1.128	2.758
Substitution protocol (yes/no)	0.000	0.379	0.300	0.479	0.028	0.511	0.280	0.931
Poly substance use	0.001	0.642	0.493	0.835	0.098	0.423	0.152	1.173
Subjects using doses higher than the AMM dose	0.001	2.041	1.328	3.138	0.004	2.255	1.300	3.913
Injection drug use	<0.0001	4.997	2.437	10.245	0.010	4.608	1.445	14.693
Intranasal use	<0.0001	3.767	2.791	5.083	0.022	2.109	1.113	3.994
Illegal obtaining	<0.0001	12.161	9.042	16.356	0.000	3.796	2.190	6.579
Benzodiazepines use	<0.0001	0.423	0.335	0.533	0.000	2.677	1.614	4.438
Heroin or speedball use	<0.0001	6.278	3.698	10.659	0.029	4.241	1.161	15.486
Cannabis use	<0.0001	2.705	2.155	3.396	0.001	2.761	1.550	4.919
Cocaine (or crack) use	<0.0001	4.356	3.170	5.986	0.004	3.301	1.474	7.392
Withdrawal symptoms from at least one of the substances	<0.0001	3.059	2.432	3.846	0.000	2.443	1.595	3.742

For the multivariate analysis: Omnibus sig *P* < 0.0001; model summary Cox and Snell R 30.8%; Hosmer–Lemeshow not significant.

## 4 Discussion

This study describes the characteristics of participants reporting dependence and/or abuse versus those reporting simple use of PAS 1 week prior to incarceration and the evolution in the trend of use between 2013 and 2022. A previous study over the period 2003–2006 used the same program in jails compared to subjects included in other structures of care [8]. Results showed that prison inmates are younger and present worse social‐economical indicators and consume more products, more illicit ones and more benzodiazepines such as flunitrazepam and clonazepam prior to imprisonment. If we compare the overall consumption data of our population with that of this previous study, we can note a lower consumption of cocaine/crack (19.6% versus 28.6%), and benzodiazepines (15.5% versus 23.1%), but a comparable level of heroin consumption (10% versus 14.8%) [8]. Despite an abundance of research on substance abuse and dependence within prison populations in France, there remains a critical gap in understanding how specific prison inmates' characteristics are associated with these issues. This lack of targeted studies hinders our ability to tailor interventions effectively, emphasising the need for more nuanced investigations into these relationships.

Yet, in our study, we aimed to evaluate the association of participant characteristics with simple use or abuse/dependence to advocate for evidence‐based abuse/dependence treatment of adults in a prison setting. We found that 85.5% of participants reported abusing/being dependent on at least one substance, which is much higher than the prevalence of substance use assessed in another French study among men prison inmates (28.7%) compared to the general population (5%) [13].

Results showed that higher rates of abuse and dependence were seen among men in comparison to women, as part of our study findings. These results should be treated with caution given the low representation of women in our study (8.4%); the prisons included were predominantly male establishments. However, this percentage is comparable to European data, which estimates the representation of women in European prisons at 5% [1]. However, research has frequently identified key differences in SUDs between males and females [14, 15] and similar results were identified in another published paper, which demonstrated that male sex was a predictor of SUD [16].

When assessing reported substances by participants, cannabis was the most reported substance over the period of our study, followed by cocaine, benzodiazepines and then heroin. Comparable prevalences were found in another study carried out in the prisons of a single region in France among 800 prison inmates, the week preceding incarceration (as in our study): cannabis (49%), cocaine (16.5%), heroin (8.9%) and benzodiazepines (15.9%) [17]. This is the same for another study carried out among Belgian prison inmates with a prevalence of use before incarceration of 40.9% for cannabis (versus 52.8% in our study), 27.5% for cocaine/crack, 13.6% for heroin and 12.2% for benzodiazepines [5]. The consumption of benzodiazepines is higher in our study (23.1%) compared to all these other studies. The use of benzodiazepines is widely prevalent in France, even as this has decreased in recent years within the general population [18]. In subjects with SUD, benzodiazepines may be used to manage withdrawal symptoms from other substances (opioids, alcohol) or anxiety‐inducing effects after stimulant use. However, the percentage of benzodiazepine consumption in this population is comparable to that found in all subjects included in the OPPIDUM survey where the use of benzodiazepines has been around 20% for several years [7, 19]. For other substances, consumption levels are comparable to those of the entire OPPIDUM survey except for cannabis, which is much higher in the population in prisons (52.8% versus 36%) [19].

In the general European population, cannabis and cocaine are the two most used illicit substances [20]. It therefore seems consistent that these three substances (cannabis, cocaine, benzodiazepines) occupy a predominant place in preincarceration use by the subjects in our study. In a recent French study, 25% of prison inmates consumed cannabis either daily or regularly (at least 10 times a month) before their incarceration [6]. Moreover, in the United States of America, cannabis was also stated as the main substance taken preincarceration in another study where 89% of the participants who reportedly consumed PAS over the previous 6 months used cannabis followed by cocaine or powder crack (11%) and heroin (8%), as reflected in our results [21]. In addition, both cocaine and opioids including heroin were reported by other mentioned studies in prisons [22]. Comparable results showed that the highest rates of use are reported for cannabis, cocaine and amphetamines, and to a lower extent heroin and other types of drugs [23].

Similarly, prison inmates report high levels of lifetime prevalence of substance use before imprisonment and increased levels of consumption, especially of heroin, cocaine and amphetamines, compared with the general population [1]. Moreover, a greater rate of participants in the group abuse and dependence also stated a history of polysubstance use, comparable to the results presented by other studies inside and outside prison settings [24].

By contrast, several variables were identified in the final model of the multivariate analysis illustrating a significant association with abuse/dependence versus simple use. Although alcohol dependence was not identified in our final model of the multivariate analysis, bivariate analysis indicated a higher rate of abuse and dependence among participants with a reported history of alcohol dependence. These results were also noted in other studies suggesting that tobacco use and relapse are largely connected with a hazardous pattern of alcohol use rather than with moderate drinking [25]. Similarly, it was stated in the literature that smoking often occurs in combination with the use of other PAS and cigarettes may become a trigger for using illicit drugs [26]. At the same time, data from 50 countries, which represent 73.5% of all prison inmates in the world, conveyed that smoking by this particular population exceeded community rates 1.04‐ to 62.6‐fold [27].

Notably, the strongest and most significant association among substances reported by participants was observed with heroin or speedball use, followed by cocaine or crack. Cannabis and benzodiazepines showed comparatively lower odds of dependence and abuse. Several studies have compared the reinforcing effects of the coadministration of cocaine and heroin, referred to commonly as “speedball”, to either drug alone [28]. The reinforcing effects of substances like heroin, cocaine and their combination (speedball), are pharmacologically driven by how these drugs interact with the brain's reward and neurotransmitter systems [29, 30]. Heroin dependence is strongly linked to its pharmacological action on opioid receptors, with active metabolites like morphine and morphine‐6‐O‐glucuronide contributing to acute effects, tolerance, physical dependence and addiction development, underlying its high addictive potential [31, 32].

Injected drug use also was shown to raise the odds of abuse and dependence approximately four and a half times higher among subjects compared to simple use; this was further noted by other studies reporting that smoking a drug or injecting it increases its addictive potential [33, 34].

A critical finding from our statistical model was the lower odds of abuse and dependence among participants on substitution treatment, showing the OST protective effect on the risk of abuse and dependence; they had 50% lower odds of abuse and dependence compared to those who were not. Substitution treatment programmes have the greatest potential to reduce injecting drug use and the resulting risk of the spread of infection [35]. Thus, providing both drug dependence treatment and harm reduction schemes in prison is therefore essential [35]. A recent study in Australia demonstrated that upon entry to prison, injecting drug use decreased but syringe sharing increased among injectors [36]. Furthermore, the provision of OST in prison is associated with a reduced prevalence of injecting drug use in prison among people with a long‐term history of intravenous drug use [37], as well as being an opportunity to engage in drug treatment in one of the highest risk and hard‐to‐reach groups [38].

To our knowledge, this is the first study to explore the association of demographic factors, history of substance use, addictive behaviour and substance use among the recently incarcerated in France. It is also the first large‐scale study to estimate the prevalence of abuse or dependence among prison inmates over an 11 ‐year period between 2013 and 2022.

### 4.1 Strengths and Limitations

To our knowledge, this study was the first to assess the association between characteristics of participants and reported abuse or dependence. One of the strengths of the OPPIDUM survey is that it collects information on PAS consumption directly from users and, in this case, from participants interviewed in a prison setting. This is also a limitation of the study because users may not fully report what they consume. Other limitations are encountered: firstly, the OPPIDUM survey described the substance by participants suffering from abuse or dependence, or on OSTs, but provided no data on the context of use, reasons, social consequences or comorbidities. The type of consumption, simple use or abuse/dependence, is based on the declaration of the participants without further assessment. Furthermore, there is no data on whether incarceration was associated with drug‐related activities or other legal cases, and the study does not allow us to identify whether participants were seen during their first or subsequent period of incarceration. One key limitation is that our sample consists of prison inmates who are in contact with addiction services, which inherently biases our findings toward individuals with higher rates of drug use prior to incarceration. This selection bias means that the prevalence of drug use observed in our study might be higher than what would be found in the general prison population.

## 5 Conclusion

Our findings address the health and social needs of prison inmates which is crucial and should be done both during and after incarceration. Variables associated with substance use are essential for appropriate prevention and management of SUD in prison inmates, considering the vulnerability of this population. More data and robust evidence are needed to identify and quantify the drug‐related needs of prison inmates during their incarceration period and even after release from prison.

## Author Contributions

**Zeinab Abbas:** acquisition, analysis and interpretation of data, writing original draft and writing – review. **Clémence Lacroix:** conception of the work, acquisition and interpretation of data, writing – review. **Elisabeth Jouve:** acquisition, analysis and interpretation of data. **Céline Eiden:** methodology, acquisition and interpretation of data, writing – review. **Joelle Micallef:** conception of the work, methodology, interpretation of data, writing – review. **Hélène Peyrière:** conception of the work, interpretation of data, writing original draft, and writing – review and editing.

## Funding

This study was not supported by any sponsor or funding sources.

## Ethics Statement

This study has been granted an exemption from requiring ethics approval and written informed consent because the OPPIDUM program collects only anonymous data. No ethics approval is requested to process anonymous data according to French law. Written informed consent from participants was not needed for the study presented in this article in line with national guidelines.

## Conflicts of Interest

The authors declare no conflicts of interest.

## Data Availability

The data are not publicly available due to privacy or ethical restrictions. Further enquiries can be directed to the corresponding author.
